# Is reduced ferredoxin the physiological electron donor for MetVF-type methylenetetrahydrofolate reductases in acetogenesis? **A hypothesis**

**DOI:** 10.1007/s10123-021-00190-0

**Published:** 2021-07-13

**Authors:** Christian Öppinger, Florian Kremp, Volker Müller

**Affiliations:** Department of Molecular Microbiology & Bioenergetics, Institute of Molecular Biosciences, Johann Wolfgang Goethe University, Max-von-Laue Str. 9, 60438 Frankfurt, Germany

**Keywords:** Methylene-tetrahydrofolate reductase, MetVF, Acetogen, Wood-Ljungdahl pathway, Acetogenesis

## Abstract

The methylene-tetrahydrofolate reductase (MTHFR) is a key enzyme in acetogenic CO_2_ fixation. The MetVF-type enzyme has been purified from four different species and the physiological electron donor was hypothesized to be reduced ferredoxin. We have purified the MTHFR from *Clostridium ljungdahlii* to apparent homogeneity. It is a dimer consisting of two of MetVF heterodimers, has 14.9 ± 0.2 mol iron per mol enzyme, 16.2 ± 1.0 mol acid-labile sulfur per mol enzyme, and contains 1.87 mol FMN per mol dimeric heterodimer. NADH and NADPH were not used as electron donor, but reduced ferredoxin was. Based on the published electron carrier specificities for *Clostridium formicoaceticum*, *Thermoanaerobacter kivui*, *Eubacterium callanderi*, and *Clostridium aceticum*, we provide evidence using metabolic models that reduced ferredoxin cannot be the physiological electron donor in vivo, since growth by acetogenesis from H_2_ + CO_2_ has a negative ATP yield. We discuss the possible basis for the discrepancy between in vitro and in vivo functions and present a model how the MetVF-type MTHFR can be incorporated into the metabolism, leading to a positive ATP yield. This model is also applicable to acetogenesis from other substrates and proves to be feasible also to the Ech-containing acetogen *T. kivui* as well as to methanol metabolism in *E. callanderi*.

**Supplementary Information:**

The online version contains supplementary material available at 10.1007/s10123-021-00190-0.

## Introduction

Acetogenic bacteria are polyphyletic and characterized by a special pathway for acetate formation from CO_2_, the Wood-Ljungdahl pathway (WLP). This two-branched linear pathway is considered an ancient pathway of CO_2_ fixation since it is the only of the known pathways for CO_2_ fixation that is coupled to net ATP synthesis, allowing the bacteria to grow lithotrophically on H_2_ + CO_2_ or CO (Martin and Russell 2007; Martin 2012). In the methyl branch, one mol of CO_2_ is reduced to formate that is then bound at the expense of ATP hydrolysis to the C_1_ carrier tetrahydrofolate (THF). Formyl-THF is dehydrated to methenyl-THF and then reduced via methylene- to methyl-THF. The methyl group condenses with a carbonyl group, derived from a second molecule of CO_2_ by action of the CO dehydrogenase/acetyl-CoA synthase (CODH/ACS), and CoA to acetyl-CoA which is further converted to acetyl-phosphate and to acetate; the latter reaction is coupled to the synthesis of ATP by substrate level phosphorylation (Drake et al., 2008; Schuchmann and Müller 2014).

How this pathway is coupled to the synthesis of net ATP has been an enigma for decades (Müller 2003), but in the last couple of years, it was demonstrated unequivocally that acetogens have a respiratory chain consisting of an ATP synthase and a respiratory redox enzyme (Schuchmann and Müller 2014). The latter is either an energy-conserving hydrogenase (Ech) or an Rnf complex; both enzymes use reduced ferredoxin (Fd_red_) as electron donor and either H^+^ (Ech) or NAD^+^ (Rnf) as acceptor (Biegel and Müller 2010; Hess et al., 2014; Müller et al., 2008; Schölmerich and Müller 2019; Westphal et al., 2018). Acetogenesis from H_2_ + CO_2_ according to:

1 $$4\;H_2+2\;{CO}_2\rightarrow{CH}_3COOH+2\;H_2O\;\left(\Delta G_0\text{'}=-95\;kJ/mol\right)$$

or carbon monoxide according to:

2 $$4\;CO+4\;H_2\rightarrow{CH}_3COOH+2\;{CO}_2\;\left(\Delta G_0^\text{'}=-165.6\;kJ/mol\right)$$

allows for the synthesis of only a fraction of an ATP, considering environmental H_2_ or CO concentrations and thus this lifestyle clearly is at the thermodynamic edge of life (Schuchmann and Müller 2014).

**Fig. 1:**
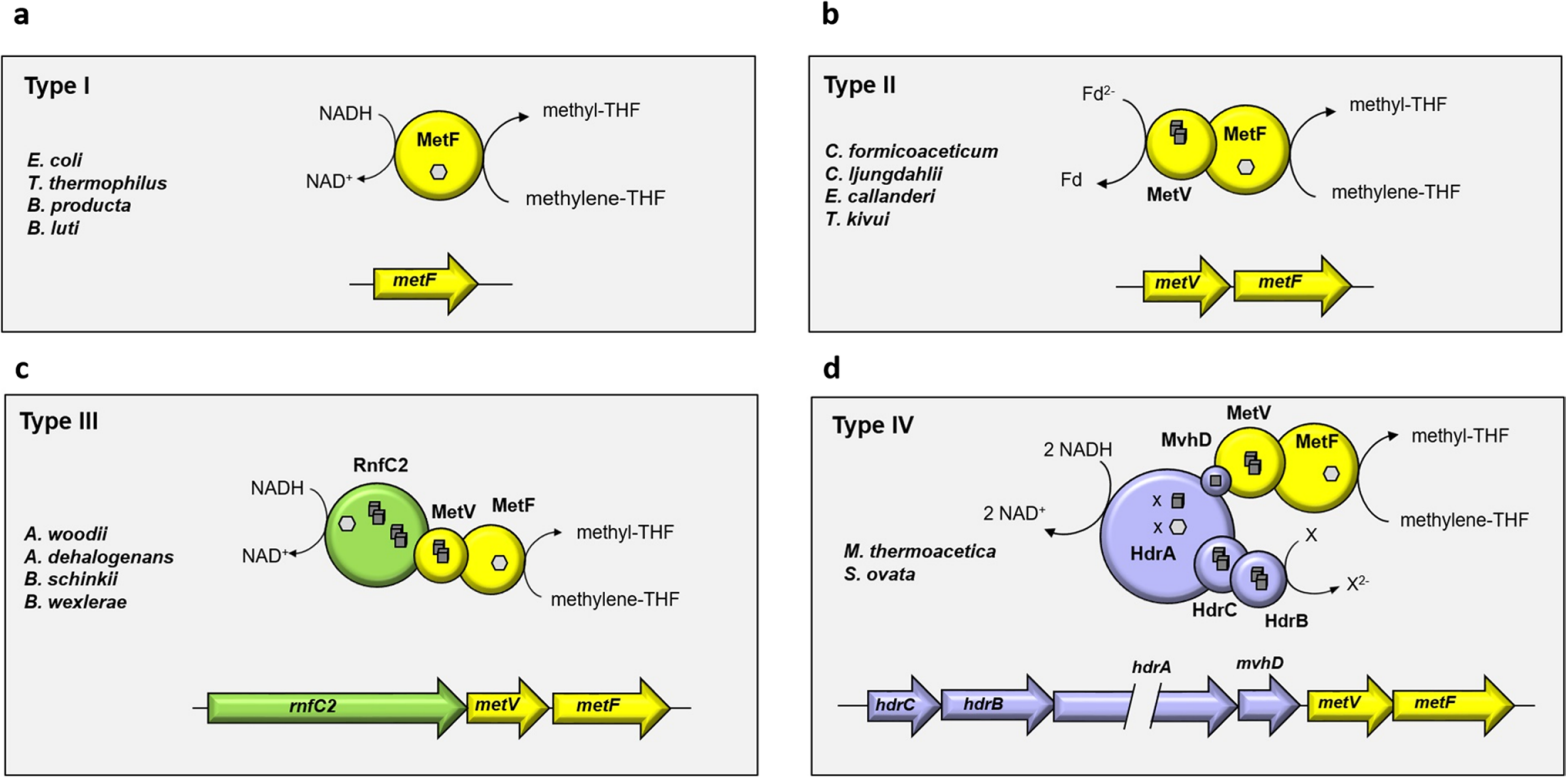
Overview of different types of methylene-THF reductases in acetogenic bacteria. Type I consists of only one subunit (MetF) that catalyzes NAD(P)H-dependent methylene-THF reduction (**a**). Type II consists of MetF and an additional, FeS and zinc-containing subunit MetV (**b**). Type II-MTHFRs were reported to use reduced ferredoxin as electron donor for methylene-THF reduction in vitro. The type III-MTHFR consists of the subunits MetV, MetF and an RnfC-like subunit (RnfC2), which harbors the NADH-binding site (**c**). Type III-MTHFRs use NADH as reducing agent. In Type IV-MTHFRs, the subunits MetV and MetF are proposed to build a complex with the heterodisulfide (Hdr) –like subunits HdrCBA and a methyl viologen reducing hydrogenase-like subunit MvhD (**d**). Type IV-MTHFRs are proposed to use NADH as electron donor for simultaneous reduction of methylene-THF and a second, still unknown electron acceptor. Arrows indicate the genetic organization of the different types. Representative species are given for each type. Hexagons represent flavins, cubes indicate [4Fe4S] clusters, and squares indicate [2Fe2S] clusters. The number of FeS clusters and flavins bound by HdrA differs between the organisms and is therefore indicated by an x

One of the energetically important reactions in acetogenesis is the reduction of methylene-tetrahydrofolate to methyl-tetrahydrofolate, catalyzed by the methylene-THF reductase. The redox couple methylene/methyl-THF has a redox potential of − 200 mV (Wohlfarth and Diekert 1991), and thus, reduction with, for example, NADH (ΔE_0_′ =  − 320 mV) is exergonic and was already in 1977 discussed to be an energy-conserving reaction in acetogenesis (Thauer et al., 1977). Unfortunately, evidence for this is still missing. MTHFRs in acetogens can be grouped in four different groups (Fig. 1). The simplest form of the MTHFR consists solely of MetF which catalyzes the irreversible reduction of methylene-THF with NADH; these are grouped in type I-MTHFRs. Classical examples of those are the MTHFRs from *E. coli*, *Thermus thermophilus*, and *Blautia producta* (Fig. 1) (Igari et al., 2011; Sheppard et al., 1999; Wohlfarth et al., 1990). Crystal structures of the enzymes from *E. coli* and *T. thermophilus* suggest a shared binding site for NAD(P)H and methylene-THF near the flavin cofactor FAD. NAD(P)H reduces the flavin and the residual NAD(P)^+^ exits the binding pocket to make way for methylene-THF, which in turn gets reduced by the enzyme-bound FADH_2_ (Lee et al., 2009; Pejchal et al., 2005). In contrast, MetF of *A. woodii*, *M. thermoacetica*, *E. callanderi*, and *Clostridium ljungdahlii* have no predicted NADH binding site (Bertsch et al., 2015; Mock et al., 2014).

In acetogenic bacteria, the MTHFR is part of the catabolic route and enzymatic activities should be much higher compared to MetF used only in anabolic routes as, for example, in *E. coli*. Type II-MTHFRs have MetV encoded next to *metF* (Fig. 1) (Clark and Ljungdahl 1984; Dietrich et al., 2021). Indeed, the additional subunit MetV has been shown to stimulate methylene-THF reduction catalyzed by MetF dramatically (Mock et al., 2014). MetV is predicted to have Zn^2+^ ions bound and to contain iron-sulfur clusters. The purified MTHFR from *Clostridium formicoaceticum* had a α_4_β_4_ composition of subunits with molecular masses of 35 and 26 kDa, presumably MetF and MetV (Clark and Ljungdahl 1984). Interestingly the electron donor for the reduction of methylene-THF was not NAD(P)H but reduced ferredoxin. The enzyme contains iron, acid-labile sulfur, and FAD whereas MetF from *E. coli* — a 33 kDa protein — only has FAD. Only reduced ferredoxin and FADH_2_ served as electron donor. The reverse reaction (oxidation of methyl-THF) was not coupled to ferredoxin reduction but only to FAD and rubredoxin reduction (Clark and Ljungdahl 1984). The authors concluded that “although both FADH_2_ and reduced ferredoxin serve as electron donors in the methylene-THF reductase reaction in *C. formicoaceticum*, we suggest that reduced ferredoxin acts under physiological conditions”. And further “Electrons could be transferred from the reduced ferredoxin to the iron-sulfur centers of the reductase, then to the enzyme-bound FAD, and finally to methylene-tetrahydrofolate” (Clark and Ljungdahl 1984). Very recently, type II-MTHFRs were purified from *Clostridium aceticum* (Wiechmann and Müller 2021), *Eubacterium callanderi* (Dietrich et al., 2021), and *Thermoanaerobacter kivui* (Katsyv et al., 2021). None used NAD(P)H as reductant for methylene-THF reduction and the enzymes from *E. callanderi* and *T. kivui* were shown to use reduced ferredoxin.

Type III-MTHFRs have an additional gene, *rnfC2*, next to *metVF*. This gene cluster is present in e.g. *Blautia schinkii*, *Blautia wexlerae*, *Acetobacterium dehalogenans*, and *A. woodii* (Fig. 1). The enzyme was purified from *A. woodii* and shown to indeed contain the three subunits RnfC2, MetF, and MetV in a 1:1:1 stoichiometry. The complex contained 2 FMN, 23.5 ± 1.2 Fe, and 24.5 ± 1.5 S which fits well to the predicted six [4Fe4S] clusters in MetV and RnfC2 (Bertsch et al., 2015). MTHFR catalyzed NADH:methyl viologen (MV) and NADH:ferricyanide oxidoreductase activity but also methylene-THF reduction with NADH as reductant. The NADH:methylene-THF oxidoreductase activity was high (248 U/mg) and not stimulated by ferredoxin. Reduction of ferredoxin, alone or in the presence of methylene-THF and NADH, was not catalyzed. MetF or MetVF were not able to catalyze the methylene-THF-dependent oxidation of NADH, but MetVF could reduce methylene-THF using reduced methyl viologen (MV_red_) as electron donor (Bertsch et al., 2015). The purified MTHFR complex catalyzed the reverse reaction, the endergonic oxidation of methyl-THF with NAD^+^ as acceptor with low rates, and this reaction could not be stimulated by reduced ferredoxin (Kremp et al., 2018). However, addition of methylene-THF dehydrogenase (Bertsch et al., 2015) stimulated the oxidation of methyl-THF to methylene-THF coupled to NAD^+^, indicating that the reaction is pulled by removing the end product. Thus, type III-MTHFRs are apparently soluble enzymes involved neither in chemiosmotic energy conservation nor in reducing ferredoxin (by electron bifurcation).

Type IV-MTHFR gene cluster do encode MetVF but also the proteins HdrCBA and MvhD (Mock et al., 2014). The complex was enriched from *M. thermoacetica* and shown to reduce methylene-THF with benzyl viologen as donor and oxidize NADH with benzyl viologen as acceptor. Experiments with heterologously produced subunits demonstrated that MetF is the site of methylene-THF reduction and HdrA the site of NADH oxidation. But in contrast to the type III-MTHFR of *A. woodii*, the complex from *M. thermoacetica* was not able to catalyze the methylene-THF-dependent oxidation of NADH (Mock et al., 2014). Since an involvement of ferredoxin could not be confirmed, the authors speculated that the MTHFR of *M. thermoacetica* conveys electron bifurcation from NADH to methylene-THF and a so far unknown second acceptor. This hypothesis is strengthened by the fact that known electron bifurcating enzymes are strictly coupled; i.e., even the energetic downhill reaction is not catalyzed in the absence of the second electron acceptor (Buckel and Thauer 2018; Müller et al., 2018; Schuchmann and Müller 2012).

In sum, type I- and III-MTHFRs are neither involved directly (as terminal electron acceptor) nor indirectly (by producing the fuel for the respiratory enzymes, reduced ferredoxin, by electron bifurcation) in energy conservation. Type IV-MTHFR may be coupled to energy conservation, either directly or indirectly, but this remains to be proven. In sharp contrast, present data suggest that type II-MTHFRs not only are not involved in energy conservation, but worse, use reduced ferredoxin as reductant for methylene-THF reduction, which seems to be a “waste” of energy. And more, this can be considered a “loss” of energy since less fuel is available for electron transport phosphorylation.

Here, we have purified a type II-MTHFR from another acetogen, *Clostridium ljungdahlii*. As the other previously purified type II-MTHFRs from *C. formicoaceticum*, *C. aceticum*, *E. callanderi*, or *T kivui*, the enzyme does not use reduced pyridine nucleotides as reductant but only artificial electron donors such as reduced viologen dyes as well as Fd_red_ (Clark and Ljungdahl 1984; Dietrich et al., 2021; Katsyv et al., 2021; Wiechmann and Müller 2021). We will present metabolic schemes for the abovementioned organisms that all come to the conclusion that a reduced ferredoxin-oxidizing MTHFR would have a negative ATP yield for acetogenesis from H_2_ + CO_2_ and thus would not allow for growth on H_2_ + CO_2_. Since the organisms grow on H_2_ + CO_2_, the observed electron flow from ferredoxin to methylene-THF may be an unspecific, unphysiological reaction. As a solution we will hypothesize a function for an NAD(P)H-independent type II-MTHFR that allows for net ATP synthesis during growth on H_2_ + CO_2_.

## Material and methods

### Cultivation of *C. ljungdahlii*

*C. ljungdahlii* was cultivated at 37 °C in carbonate-buffered complex media (DSMZ 879) as described before (Tanner et al., 1993), but Na_2_S was omitted. 28 mM fructose was added as a carbon and an electron source.

### Cell harvest and cytosol preparation

Cell harvest and preparation of cytoplasm was performed under strictly anoxic conditions as described before for *A. woodii* (Bertsch et al., 2015).

### Purification of the methylene-THF reductase

50 ml of cytoplasm (1290 mg) was applied to fast protein liquid chromatography on Q-sepharose high-performance (GE Healthcare, Chicago, IL, USA) previously equilibrated with buffer A (50 mM Tris–HCl, 20 mM MgSO_4_, 20% glycerol, 2 mM DTE, 4 µM resazurin, pH 7.6) at a flow rate of 2 ml/min. Proteins were eluted using a linear gradient from (0–25%) of buffer B (= buffer A + 1 M NaCl). The protein fractions with MV_red_:methylene-THF oxidoreductase activity were pooled and incubated with ammonium sulfate to a concentration of 2.4 M. Precipitated proteins were pelleted by centrifugation and the supernatant was applied to hydrophobic interaction chromatography on a Phenyl-sepharose HP column (GE Healthcare, Chicago, IL, USA) equilibrated with buffer C (= buffer A + 2.4 M (NH_4_)_2_SO_4_). The column was washed with 35% buffer A and the MTHFR was eluted with a linear gradient from 35–75% buffer A. The MTHFR-containing fractions were pooled and concentrated to 0.5 ml using Vivaspin ultrafiltration tubes (10 kDa cutoff, Sartorius Stedim Biotech GmbH, Germany). The pooled and concentrated fractions were applied to size exclusion chromatography on Superdex® 200 Increase (10/300 GL prepacked column, GE Healthcare, Chicago, IL, USA) equilibrated with buffer D (= buffer A + 250 mM NaCl) and eluted with a flow rate of 0.25 ml/min. The purified MTHFR eluted in a single Peak at around 12.9 ml and was stored at 4 °C.

### Enzymatic assays

If not stated otherwise, the assays were performed at 37 °C in phosphate buffer (50 mM NaP_i_, pH 7, 2 mM DTE). For the determination of the pH optimum, a combined buffer (50 mM MES, MOPS, Tris, CHES, CAPS, 2 mM DTE) with different pH values was used. During the purification procedure, the MTHFR was routinely measured with prereduced MV as described before (Dietrich et al., 2021) at 24 °C. If Fd_red_ was used as electron donor, methylene-THF was synthesized from formaldehyde and THF as described before (Bertsch et al., 2015) and 30 µM ferredoxin was added to an anaerobic cuvette filled with phosphate buffer. Na_2_S_2_O_4_ was added until ferredoxin was reduced to 90% and the reaction was started by addition of the enzyme. The oxidation of Fd_red_ was recorded at 430 nm (ε_ox-red_ = 13.1 mM^−1^ × cm^−1^).

### Analytical methods

The native molecular mass was determined by gel filtration on Superdex® 200 Increase using the “high- “ and “low-molecular weight” kits (GE, Healthcare, Sweden) for calibration of the column. The protein concentration was determined according to the method of Bradford (1976). Proteins were separated in a denaturating SDS-PAGE according to Laemmli (1970), using 12% polyacrylamide gels. Gels were stained with Coomassie brilliant blue G250. The iron and sulfur content was determined colorimetrically according to Fish (1988) and Beinert (1982). The nature of the flavin and the flavin content were determined as described before Bertsch et al., (2013).

## Results and discussion

### The methylene-THF reductase is of the MetV/MetF type

**Fig. 2:**
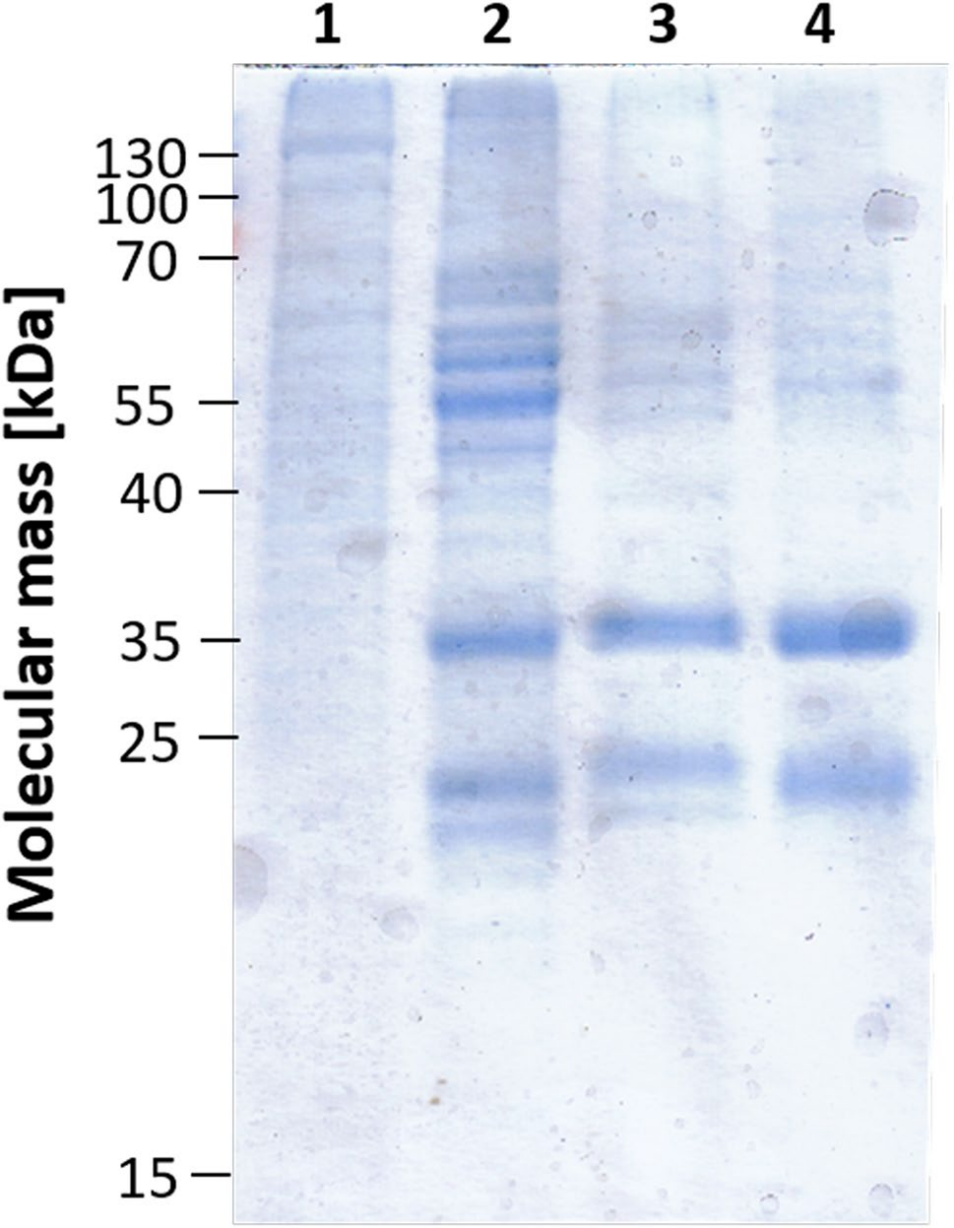
Purification of the MetVF-type MTHFR from *C. ljungdahlii.* The MTHFR from *C. lungdahlii* was purified in 3 chromatographic steps. 10 µg protein of each step was separated in a denaturating SDS-PAGE and stained with Coomassie brilliant blue. 1, cytoplasm; 2, Q-sepharose; 3, Phenyl-sepharose; 4, Superdex® 200 Increase

The genome of *C. ljungdahlii* does encode neither RnfC2 nor HdrCBAMvhD. The genes next to *metVF* are *folD* and *lpdA*, encoding a bifunctional methylene-THF dehydrogenase/methylene-THF cyclohydrolase and lipoamide dehydrogenase (Supplementary Fig. 1). For the purification of the MTHFR, *C. ljungdahlii* was grown in 20 l medium with fructose (28 mM) as carbon and electron source. After the culture reached late exponential growth phase (OD_600_ of ~ 2–2.5), cells were harvested (40–50 g cells) and disrupted by passing them through a French pressure cell. Cell debris and membranes were removed and the cytoplasm was used for the further purification. Since the MTHFR is able to use artificial electron donors for the reduction of methylene-THF, we used reduced methyl viologen as electron donor and followed the MV_red_:methylene-THF oxidoreductase activity during the purification procedure. The MTHFR activity was enriched 57.6-fold from 2.7 U/mg to 156.1 U/mg by three chromatographic steps on Q-sepharose, Phenyl-sepharose, and Superdex® 200 Increase (Table 1). To verify the success of the purification, protein samples of each purification step were separated in a denaturating SDS-PAGE (Fig. 2) and only two major proteins with molecular masses of 35 and 23 kDa became visible after staining with Coomassie brilliant blue. The observed molecular masses of the purified protein fit quite well to the predicted size of MetV (23 kDa) and MetF (33 kDa), and further the identity of the proteins was confirmed by MADLI-TOF analysis.

**Table 1:** Purification of the MTHFR from *C. ljungdahlii*

	Protein [mg]	MV_red_:methylene-THF OR [U*/mg]	Total activity [U]	Yield [%]	Purification [x-fold]
Cytoplasm	1297.8	2.7	3504	100.00	1.0
Q-sepharose	14.6	119.2	1738	49.6	4.39
Phenyl-sepharose	8.4	128.4	1079	30.8	47.3
Superdex 200	3.38	156.1	528	15.1	57.6

^*^1 Unit is defined as 2 µmol methyl viologen oxidized per minute

### Basic characteristics of the MetVF-type MTHFR

**Fig. 3:**
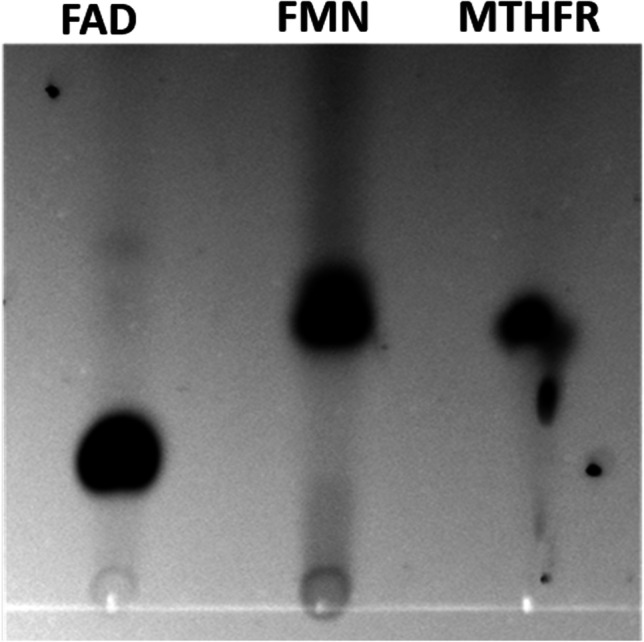
Thin layer chromatography of the flavins extracted from MTHFR. Flavins of ~ 0.5 nmol MTHFR were separated on a TLC plate using 60% [v/v] n-butanol, 15% [v/v] glacial acetic acid and 25% [v/v] H_2_O as the mobile phase. 0.5 nmol of FAD and FMN was used as standards

The native molecular mass of the MTHFR was determined to be ~ 106 kDa by analytical gelfiltration on Superdex® 200 Increase (Supplementary Fig. 2), which fits to the predicted size of a dimer consisting of two MetVF heterodimers (112 kDa). Furthermore, the MTHFR was found to contain 14.9 ± 0.2 mol Fe/mol (MetVF)_2_ and 16.2 ± 1.0 mol S/mol (MetVF)_2_, which is in accordance to the predicted two 4Fe-4S-clusters in MetV. MetF is predicted to bind a flavin and indeed we identified FMN as the flavin (Fig. 3), which is bound by the MTHFR in a stoichiometry of 1.87 mol FMN/mol (MetVF)_2_. Next we tested the enzyme’s ability to use pyridine nucleotides as electron donor for methylene-THF reduction, but no activity was observed with either NADH or NADPH. Since the MTHFR was proposed to use the mechanism of flavin-based electron bifurcation (Köpke et al., 2010), we checked if NAD(P)H serves as electron donors when Fd was added as additional electron acceptor, but also neither NAD(P)H oxidation nor ferredoxin reduction could be observed. Like the before mentioned type II-MTHFRs, the enzyme from *C. ljungdahlii* used reduced ferredoxin as electron donor. The Fd_red_:methylene-THF oxidoreductase activity was highest at a temperature of 37 °C at a pH of 7 (34.7 U/mg). The activity was relatively stable between 37 and 50 °C but decreased to 56% at 20 °C and 23% at 80 °C (Supplementary Fig. 3a). At pH values other than 7, the MTHFR was significantly less active with only 2% activity left at pH 5 and 6% activity left at pH 9 (Supplementary Fig. 3b). Half maximal activities were found at a methylene-THF concentration of 70 µM and a Fd_red_ concentration of 9 µM (Supplementary Figs. 4a and 4b).

### *Carbon and electron flow during acetogenesis from H*_*2*_ + *CO*_*2*_* or CO*

**Fig. 4:**
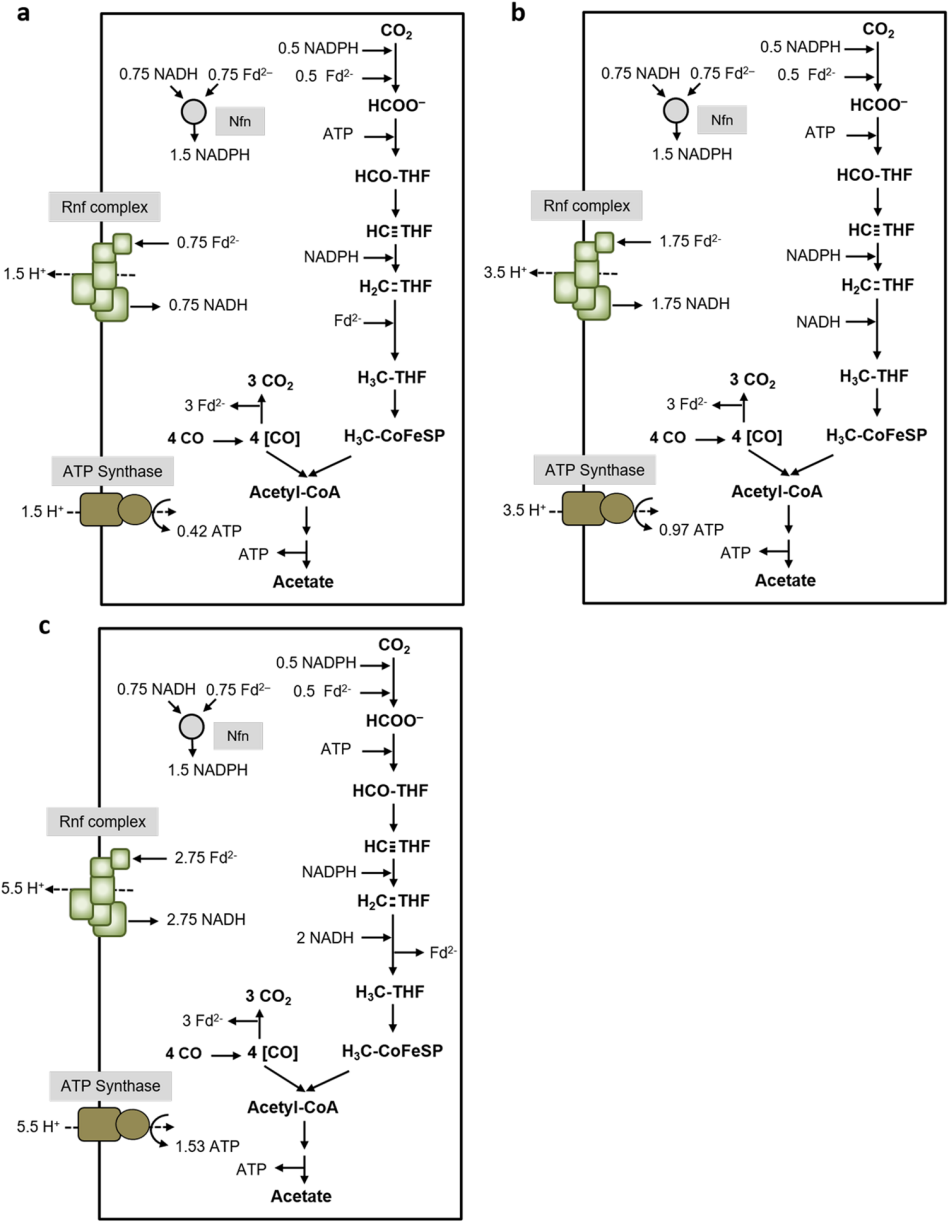
Biochemistry and bioenergetics of acetogenesis from CO in *C. ljungdahlii*. Depending on the electron donor for methylene-THF reduction, different amounts of ATP can be synthesized. In **a**, the methylene-THF reductase uses Fd_red_ as electron donor as it was observed in vitro*.* In **b**, we assume NADH or a compound with a similar redox potential as electron donor for the MTHFR, which increases the ATP yield. In **c**, we assume a MTHFR that uses NADH or a compound with a similar redox potential for simultaneous reduction of Fd and methylene-THF, which again increases the theoretical ATP yield. Assumed stoichiometries: H^+^/ATP = 3.6 (ATP synthase) and 2 H^+^/2 e^−^ (Rnf). For description of the pathway, see text

The data described above clearly demonstrate Fd_red_ as the reductant for methylene-THF reduction. But how does that fit to the path of electrons and does that allow for net ATP synthesis? The redox carriers involved in the WLP in *C. ljungdahlii* are reduced ferredoxin and NADPH in the reduction of CO_2_ to formate, NADPH in the reduction of methenyl-THF to methyl-THF, and reduced ferredoxin for the reduction of CO_2_ to CO (Wang et al., 2013). With CO as carbon source, CO is oxidized by CODH to CO_2_ with concomitant reduction of ferredoxin. 1.5 mol of reduced ferredoxin is oxidized by the methylene-THF reductase, and the formate dehydrogenase. Under these conditions, it is not the hydrogenase/formate dehydrogenase complex that catalyzes CO_2_ reduction to formate since the hydrogenase is inhibited by CO (Wang et al., 2013); instead, only the electron bifurcating formate dehydrogenase is active using reduced ferredoxin and NADPH as reductant for CO_2_ reduction to formate. The NADPH required for the methylene-THF dehydrogenase is provided by the transhydrogenase Nfn (Wang et al., 2013) that requires NADH, reduced by ferredoxin as catalyzed by the Rnf complex; this reaction leads to the generation of an electrical field driving ATP synthesis. For the ATP synthase, a H^+^/ATP stoichiometry of 3.6 is assumed (based on a number of 11 *c* subunits in the *c*-ring of the ATP synthase of *C. paradoxum* (Ferguson et al., 2006; Meier et al., 2006)), for the Rnf complex a H^+^/2 e^−^ stoichiometry of 2 (Schuchmann and Müller 2014). This sums up to a positive ATP yield of 0.42 ATP/acetate (Fig. 4a) and thus allows growth on CO. If we assume NADH (or a compound with a similar redox potential as reductant, without electron bifurcation) as electron donor for methylene-THF reduction, the ATP yield increases to 0.97 ATP/acetate (Fig. 4b), and with an electron bifurcating MTHFR that uses NADH as reductant and methylene-THF and ferredoxin as oxidant, the ATP yield is even better (Fig. 4c).

**Fig. 5:**
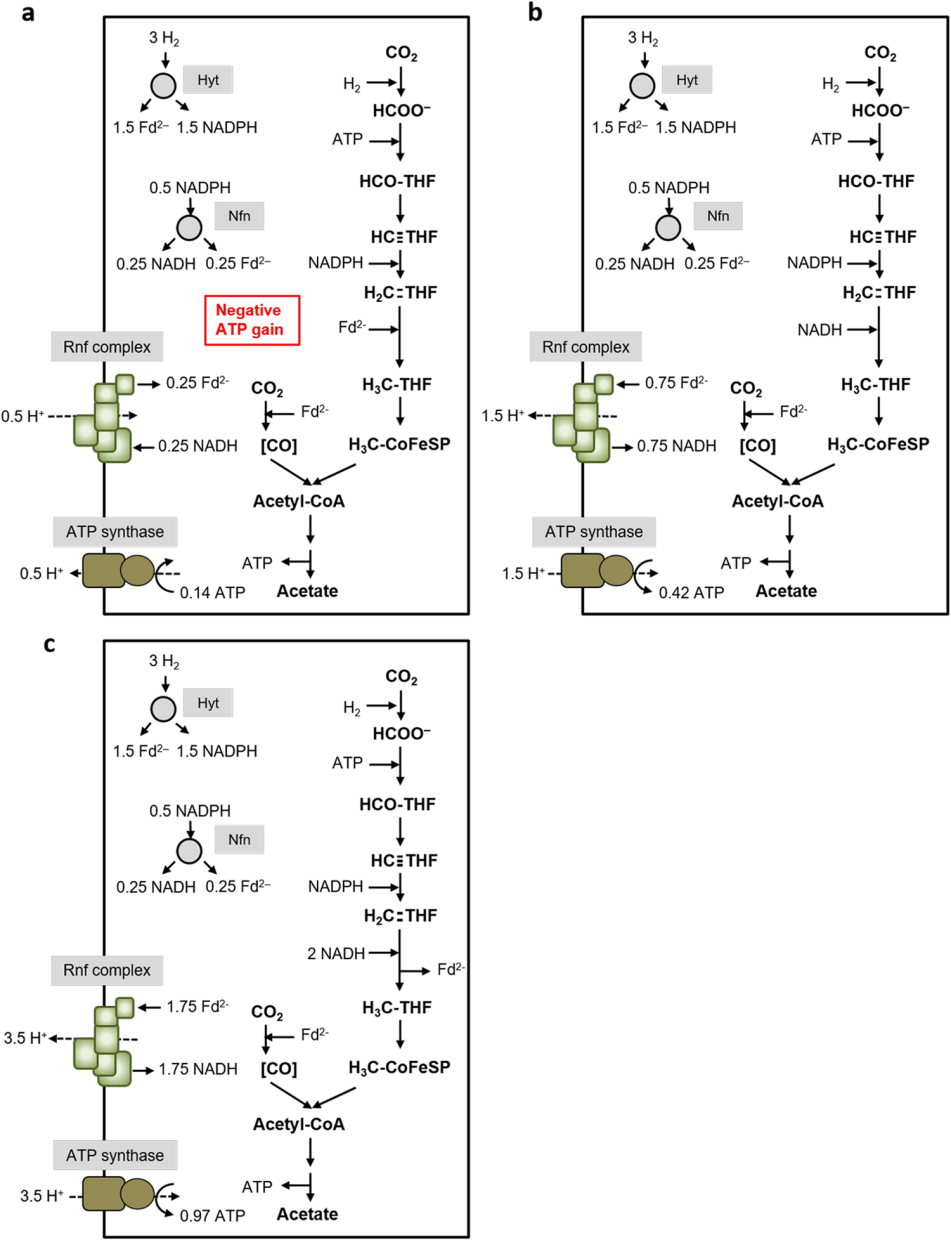
Biochemistry and bioenergetics of acetogenesis from H_2_ + CO_2_ in *C. ljungdahlii*. Depending on the electron donor for methylene-THF reduction, different amounts of ATP can be synthesized during acetogenesis from H_2_ + CO_2_. In **a**, the methylene-THF reductase uses Fd_red_ as electron donor as it was observed in vitro*.* Please note that the ATP gain in this scenario is negative. In **b**, we assume NADH or a compound with a similar redox potential as electron donor for the MTHFR, which increases the ATP yield. In **c**, we assume a MTHFR that uses NADH or a compound with a similar redox potential for simultaneous reduction of Fd and methylene-THF, which again increases the theoretical ATP yield. Assumed stoichiometries: H^+^/ATP = 3.6 (ATP synthase) and 2 H^+^/2 e^−^ (Rnf). For description of the pathway, see text

Thus, growth on CO with a reduced ferredoxin-dependent methylene-THF reductase is possible. However, this is in sharp contrast to growth on H_2_ + CO_2_. Under these conditions, the formate dehydrogenase/hydrogenase complex is active oxidizing 1 mol of hydrogen for the reduction of CO_2_ to formate. Oxidation of 3 mol H_2_ by the electron-bifurcating, NADP^+^ and ferredoxin reducing hydrogenase gives 1.5 mol NADPH and 1.5 mol reduced ferredoxin. 1 mole of ferredoxin is consumed by the CODH/ACS reaction and 1 mol NADPH by the methylene-THF dehydrogenase. If reduced ferredoxin is the in vivo reductant for methylene-THF, the excess 0.5 mol NADPH is converted by the transhydrogenase Nfn to 0.25 mol reduced ferredoxin and 0.25 mol NADH. The latter is used to reduce ferredoxin, catalyzed by the Rnf complex, and this endergonic reaction is driven by proton influx; the proton gradient is established by ATP hydrolysis. Altogether, this sums up to a negative ATP yield of − 0.14 ATP/acetate (Fig. 5a)! Since *C. ljungdahlii* grows on H_2_ + CO_2_, ferredoxin cannot be the reductant for methylene-THF reduction in vivo! If we assume NADH (or a compound with a similar redox potential) as reductant (without electron bifurcation), the ATP yield is positive, with 0.42 ATP/acetate (Fig. 5b). With an electron bifurcating MTHFR that uses NADH as reductant and methylene-THF and ferredoxin as oxidant, the ATP yield is even better (Fig. 5c). Therefore, reduced ferredoxin is excluded as electron donor for methylene-THF reduction. *C. ljungdahlii* is known to produce some ethanol alongside acetate most probably using the AOR pathway. With ethanol as product and Fd_red_ as electron donor for the MetVF-type MTHFR, the ATP yield becomes slightly positive (0.14 ATP/ethanol). However, since only minor amounts of ethanol are produced, this does not result in an overall positive ATP yield.

### Solutions to the energetic dilemma

So far, only reduced ferredoxin was identified as physiologically possible reductant for the MTHFR. However, the use of reduced ferredoxin in the reaction does not allow for net ATP synthesis during acetogenesis from H_2_ + CO_2_, an absolute prerequisite for growth. Thus, reduced ferredoxin must be excluded as reductant for methylene-THF in vivo*.* But how can we explain the observed oxidation of reduced ferredoxin with methylene-THF? It should be remembered that Clark and Ljungdahl (1984) demonstrated activity also with FADH_2_ and in the reverse reaction only with FAD but *not* with ferredoxin. Nonetheless, the authors favored ferredoxin as electron carrier. It should be kept in mind that in these enzymatic assays, ferredoxin is reduced enzymatically, by, for example, CODH and CO (Dietrich et al., 2021). But under these conditions, flavins are also reduced, without the involvement of ferredoxins. Typically, methylene-THF reductases are assayed in the presence of flavins (Bertsch et al., 2015; Mock et al., 2014; Wang et al., 2013), and, therefore, it cannot be excluded that flavins are the ultimate electron donor rather than reduced ferredoxin. Indeed, the activity in the absence of flavins is much lower and the residual activity observed may be due to the flavins still bound to the enzyme. So the interpretation of the observation is most likely not correct and reduced ferredoxin is not the electron donor in vivo. One solution to the dilemma is to assume that the use of reduced ferredoxin is an unspecific reaction.

**Fig. 6:**
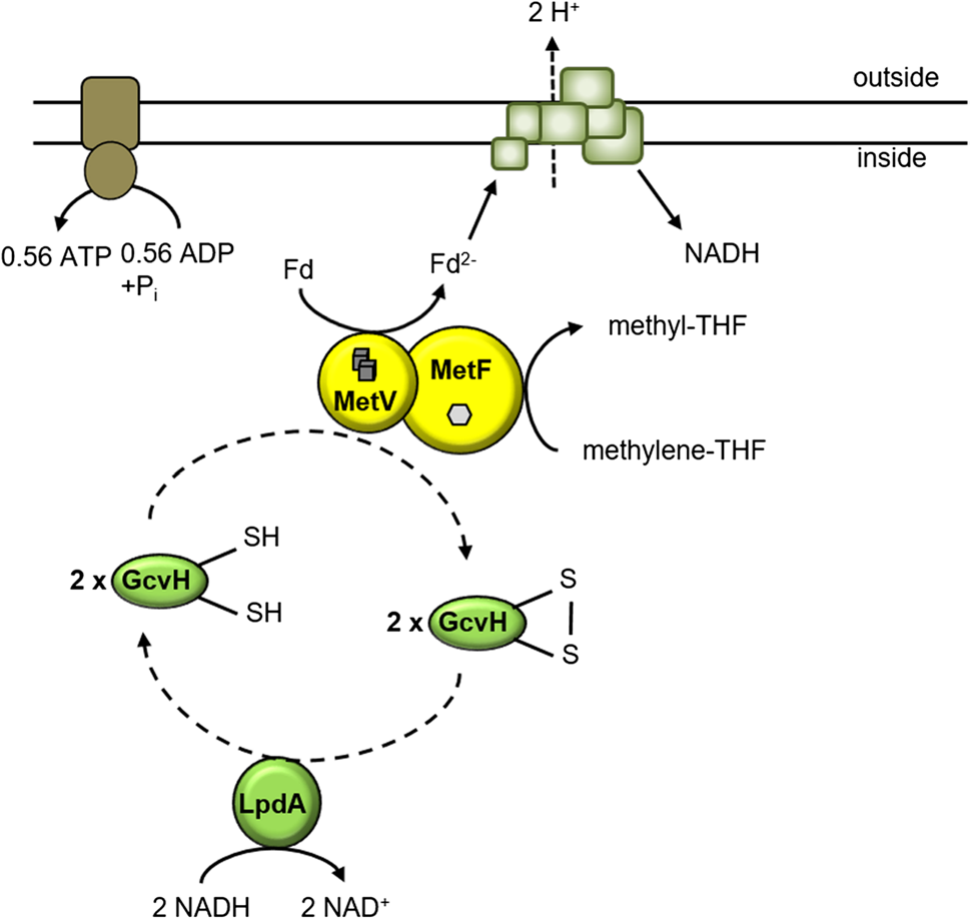
Model of electron bifurcation by the type II MTHFR involving LpdA and GcvH. GcvH is the electron donor for simultaneous reduction of methylene-THF and Fd by the MTHFR. Fd_red_ can subsequently be used for NAD^+^ reduction by the Rnf complex thereby generating an H^+^ gradient across the membrane. Resulting NADH can be used (together with a second mol NADH) for regeneration of the dihydrolipoamide cofactor of GcvH. The H^+^ gradient is used for ATP generation by the ATP synthase

But then, an alternative reductant is required. NADH could be involved, but so far, neither NADH-dependent methylene-THF reduction nor NADH:viologen oxidoreductase reaction could be observed, for any of the purified type II-enzymes (Clark and Ljungdahl 1984; Dietrich et al., 2021; Katsyv et al., 2021; Wiechmann and Müller 2021)! Moreover, neither MetF nor MetV have a conserved NADH binding site; this is likely why type III-MTHFRs have evolved RnfC2 as NADH binding/oxidizing module connected to MetVF (Bertsch et al., 2015). This makes NADH as reductant in type II-MTHFRs highly unlikely. Alternatives could be i.e. a lipoic acid binding cofactor, flavodoxin, thioredoxin, or rubredoxin. If we, nevertheless, assume NADH as reductant and furthermore assume electron bifurcation by the MetVF complex, the overall energetics gets positive (see Fig. 5b and c) but what could be the second electron acceptor? Ferredoxin reduction with NADH as reductant and methylene-THF as co-oxidant has never been detected, and again, there is no NADH binding site in MetVF. How about a different donor/acceptor pair? Next to *metVF* is a gene encoding a lipoamide dehydrogenase (LpdA) (Supplementary Fig. 1), and three genes encoding GcvH, a lipoic acid–containing protein, can also be found in the genome. One scenario could be that the oxidized form of GcvH (E_0_′[lipoic acid] =  − 325 mV (Sponar and Jirsa 1958)) is the co-oxidant alongside methylene-THF and reduced ferredoxin the electron donor. Reduced GcvH could be oxidized by LpdA with concomitant reduction of NADH. In sum, this would result in 2 mol reduced ferredoxin oxidized, coupled to the reduction of NAD^+^ and methylene-THF. Both electron transfer reactions are exergonic, and therefore, this scenario can be excluded. However, if we assume reduced GcvH as donor and ferredoxin and methylene-THF as acceptor, this becomes feasible for an electron bifurcating reaction. Oxidized GcvH could then be reduced by LpdA with NADH as reductant (Fig. 6). This would also explain the oxidation of reduced ferredoxin (the reverse reaction: reduction of oxidized GcvH with Fd_red_). However, a major drawback of this scenario is that any electron bifurcating reaction known to date requires the second acceptor for activity, i.e., does *not* show uncoupling. In this case, this must be methyl-THF. Unfortunately, oxidation of reduced ferredoxin proceeds in the absence of second acceptor. Moreover *lpdA* and *gcvH *are also found next to genes encoding type III-MTHFRs. Therefore, this scenario also seems highly unlikely.

### The hypothesis

**Fig. 7:**
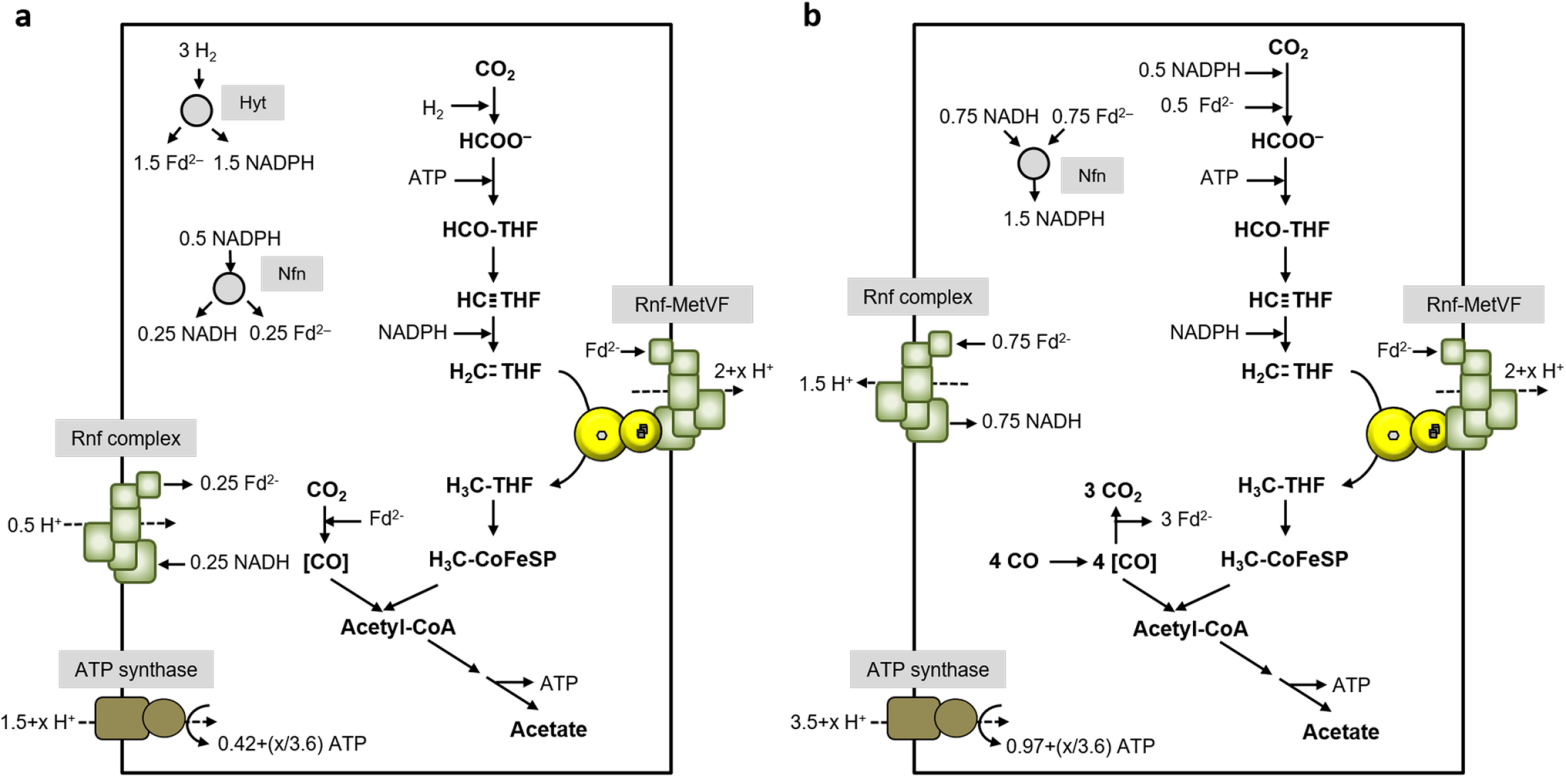
Model of Rnf-coupled methylene-THF reduction in acetogenesis of *C. ljungdahlii.* Acetate is formed from H_2_ + CO_2_ (**a**) or CO (**b**). In contrast to the model of Fig. 5a, acetogenesis from H_2_ + CO_2_ is feasible if we assume methylene-THF reduction by a membrane-coupled Rnf-MetVF complex. Assumed stoichiometries: H^+^/ATP = 3.6 (ATP synthase), 2 H^+^/2 e^−^ (Rnf) and 2 + x H^+^/2 e^−^ (Rnf-MetVF). ATP gain might be enhanced by x/3.6 ATP. For description, see text

Methylene-THF reductases have always been found in the soluble fraction but to some extent also in the membrane fraction (Hugenholtz et al., 1987; Wohlfarth et al., 1990). This is not in strong favor of a membrane localization but also does not rule out a membrane attachment, for example by a loose association with membrane proteins. Interestingly, RnfC2 of type III-MTHFRs has a MetV domain N-terminal to the RnfC domain and was hypothesized to connect the type III-MTHFR to the Rnf complex, by docking RnfC2 instead of RnfC to the complex (Poehlein et al., 2012). If we now electrically connect the Rnf complex to the type II-MTHFR, acetogenesis from H_2_ + CO_2_ yields net ATP for growth. 3 mol of hydrogen are oxidized by HytA-E yielding 1.5 mol reduced ferredoxin and 1.5 mol of NADPH. 1 mol reduced ferredoxin goes into the CODH/ACS; 1 mol of NADPH goes into the methylene-THF dehydrogenase. The remaining 0.5 mol of NADPH is oxidized by the Nfn complex, giving 0.25 mol reduced ferredoxin and 0.25 mol of NADH. The latter is oxidized by the “normal” Rnf complex to reduce ferredoxin, at the expense of proton influx. This gives, altogether, 1 mol of reduced ferredoxin that is oxidized by the Rnf-MetVF complex, thereby reducing methylene-THF to methyl-THF. In the previous chapter, we have assumed the H^+^/2 e^−^ stoichiometry of Fd_red_:NAD^+^ oxidoreductase, as catalyzed by Rnf, to 2. However, the free energy change of Fd_red_:methylene-THF oxidoreductase activity is much higher (ΔG_0_′ =  − 48.3 kJ/mol, compared to ΔG_0_′ =  − 25 kJ/mol; based on redox potentials of − 450 mV for Fd/Fd_red_, − 320 mV for NAD^+^/NADH, and − 200 mV for the methylene-THF/methyl-THF couple). Although a higher ΔG_0_′ value does not automatically cause a higher number of pumped protons, this could be the case. Therefore, we indicate “2 + x” ions for this reaction in our models. When we calculate with 2 H^+^/2 e^−^ and 3.6 H^+^/ATP, this sums up to 0.42 ATP/acetate (Fig. 7a). Acetogenesis from CO shows an even higher ATP yield of 0.97 ATP/acetate (Fig. 7b). Next, we analyzed whether a membrane-bound Fd_red_:methylene-THF oxidoreductase would also allow growth of Ech-containing acetogens, such as *T. kivui*.

**Fig. 8:**
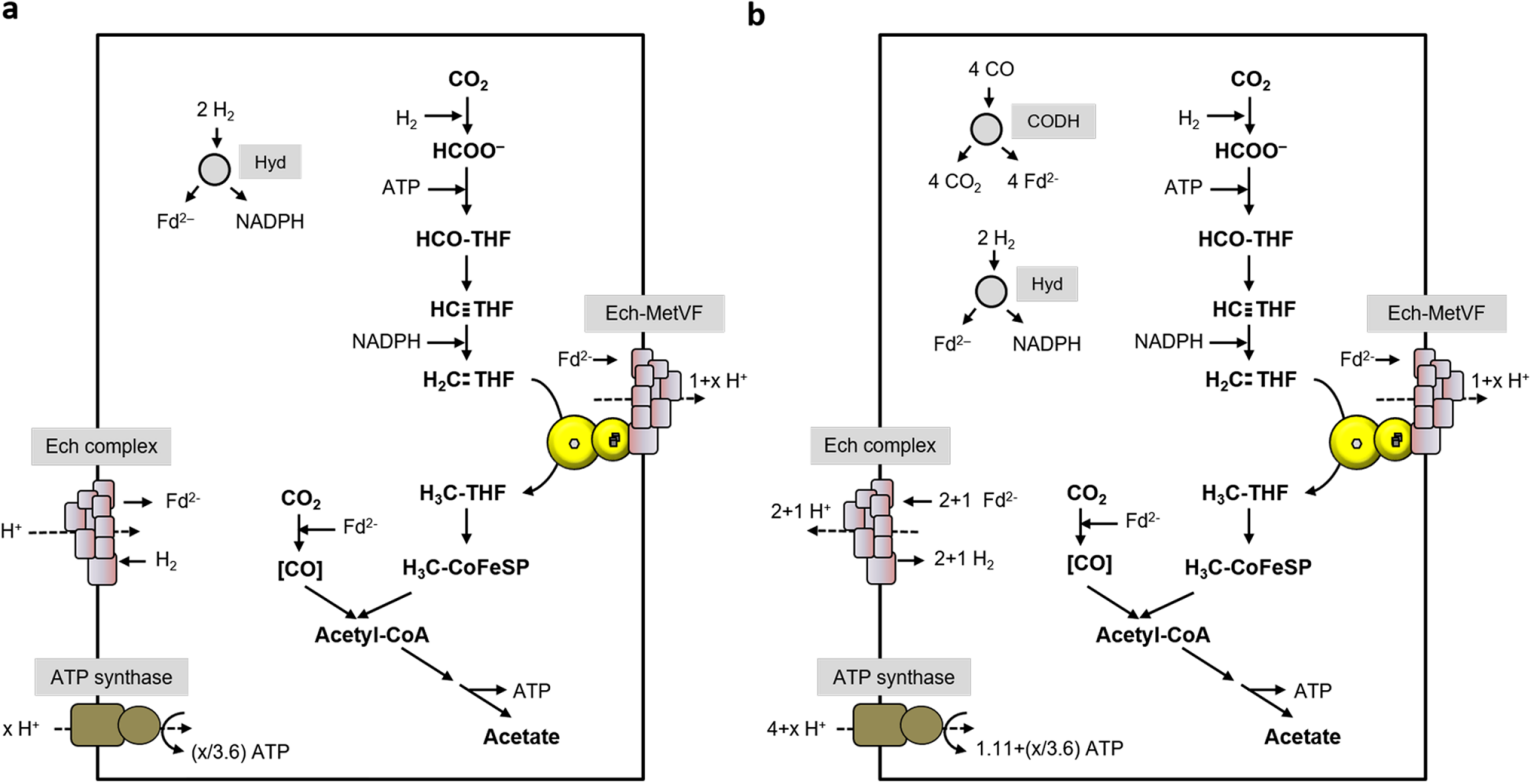
Model of Ech-coupled methylene-THF reduction in acetogenesis of *T. kivui.* Acetate is formed from H_2_ + CO_2_ (**a**) or CO (**b**). Acetogenesis from H_2_ + CO_2_ is feasible if we assume methylene-THF reduction by a membrane-coupled Ech-MetVF complex, which pumps 1 + x H^+^/2 e^−^ across the membrane. Assumed stoichiometries: H^+^/ATP = 3.6 (ATP synthase), 1 H^+^/2 e^−^ (Ech) and 1 + x H^+^/2 e^−^ (Ech-MetVF). ATP gain might be enhanced by x/3.6 ATP. For description, see text

*T. kivui* is identical to *C. ljungdahlii* with respect to the WLP; it has an electron bifurcating, ferredoxin and NADP^+^ reducing hydrogenase (Katsyv et al., 2021); it uses hydrogen as reductant for the first reaction (Schwarz et al., 2018), NADPH for the third (Katsyv et al., 2021), and reduced ferredoxin for the CODH reaction. However, it does not have an Rnf complex, but Ech (Hess et al., 2014). Because of the thermodynamics, we assume a H^+^/2 e^−^ stoichiometry of 1 for Fd_red_:H^+^ oxidoreductases like the Ech complex, but as described above, the free energy change is drastically increased, if methylene-THF serves as final electron acceptor (ΔG_0_′ =  − 48.3 kJ/mol, compared to ΔG_0_′ =  − 6.9 kJ/mol, based on a redox potential of − 414 mV for H^+^/H_2_). Therefore, we will indicate “1 + x” per methylene-THF reduced in the Ech-MetVF complex catalyzed reaction. As in *C. ljungdahlii*, CO_2_ is reduced with molecular hydrogen. 2 mol H_2 _are oxidized by the bifurcating hydrogenase, thereby reducing 1 mol NADP^+^ and 1 mol Fd, which are used for methenyl-THF reduction and CO_2_ reduction, respectively. 1 mol of hydrogen goes into CO_2_ reduction to formate. The missing 1 mol of reduced ferredoxin, for the Fd_red_:methylene-THF reduction, is provided by Ech that catalyzes a proton-driven reverse electron flow to allow ferredoxin reduction with H_2_. In this scenario, net ATP is only synthesized if the number of protons exported is higher than the number of protons imported (Fig. 8a). In contrast, acetogenesis using CO as carbon and electron source is possible with an ATP/acetate ratio of 1.11 (Fig. 8b).

**Fig. 9:**
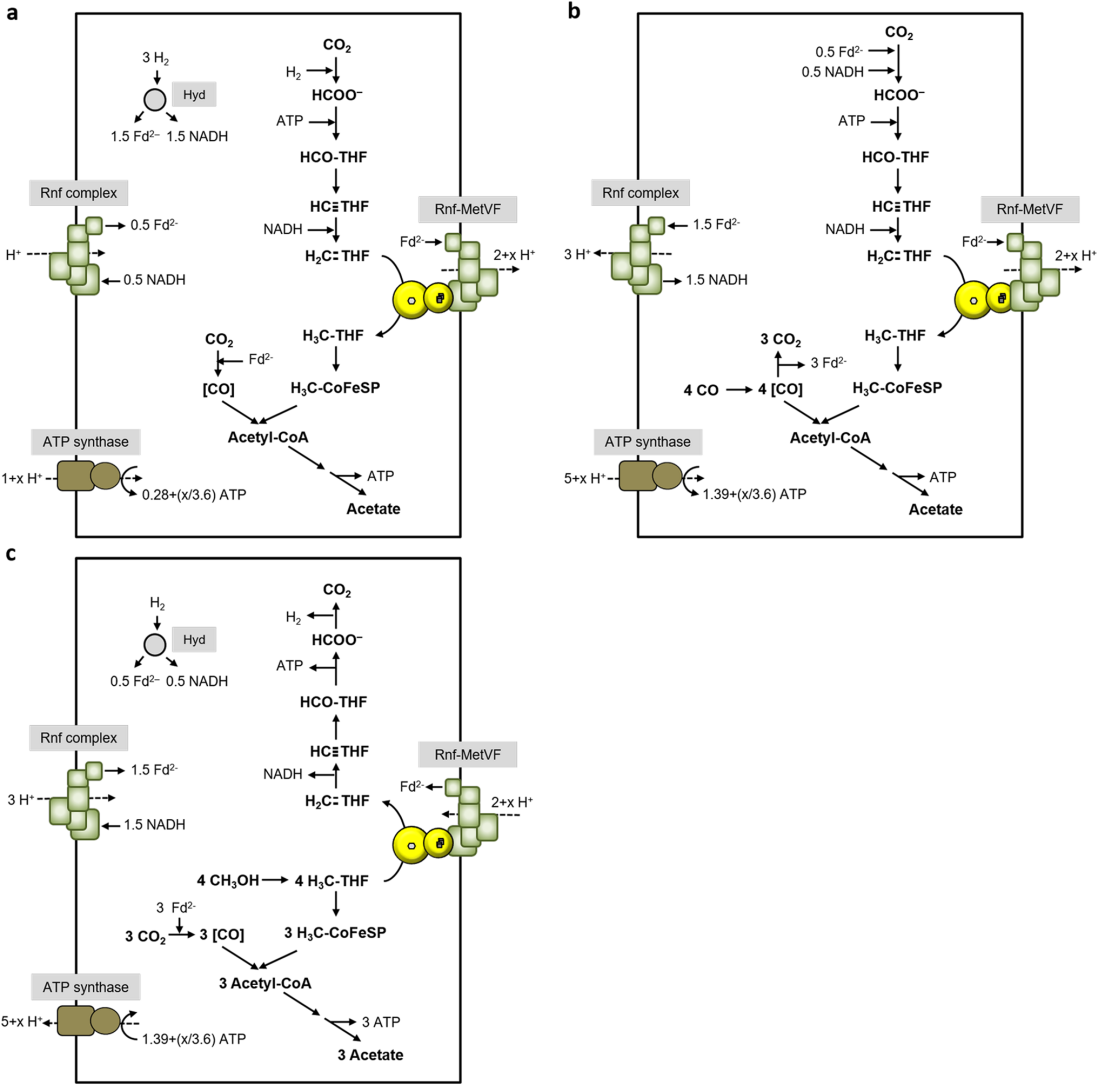
Model of Rnf-coupled methylene-THF reduction in acetogenesis of *E. callanderi.* Acetate is formed from H_2_ + CO_2_ (**a**), CO (**b**), or methanol (**c**). Acetogenesis from H_2_ + CO_2_ is feasible if we assume methylene-THF reduction by a membrane-coupled Rnf-MetVF complex. This scenario additionally explains how the energetic barrier in methyl-group oxidation during methylotrophic growth is overcome. Assumed stoichiometries: H^+^/ATP = 3.6 (ATP synthase), 2 H^+^/2 e^−^ (Rnf) and 2 + x H^+^/2 e^−^ (Rnf-MetVF). ATP gain might be enhanced (**a** and **b**)/reduced (**c**) by x/3.6 ATP. For description, see text

*E. callanderi* KIST612 grows on H_2_ + CO_2_ but also well on CO (Chang et al., 1997). It has an electron bifurcating, NAD^+^ and ferredoxin reducing hydrogenase, which can also interact with a formate dehydrogenase to use H_2_ as reductant for CO_2_ reduction to formate (Dietrich et al., 2021). Further, it has an NADH-dependent methylene-THF dehydrogenase and a MetVF-type MTHFR as it is found in *C. ljungdahlii* (Dietrich et al., 2021). According to Fig. 9a, 0.28 ATP/acetate are generated with a membrane bound Fd_red_:methylene-THF oxidoreductase when grown on H_2_ + CO_2_. When growing on CO, the electrons originate from oxidation of 3 mol CO to CO_2_. In sum, 5 H^+^ drive ATP synthesis with a yield of 1.39 ATP/acetate, if we calculate with 1 H^+^ pumped/Fd_red_ oxidized by the membrane-bound Rnf-MetVF complex (Fig. 9b).

*E. callanderi* is also known as methylotrophic organism, which grows well on methanol (Dietrich et al., 2021; Kremp and Müller 2021). When methanol is utilized, the methyl groups of 4 mol methanol are transferred to THF by the methyltransferase system. Since one methyl group has to be oxidized to generate electrons (1 mol H_2_, NADH and Fd_red_, respectively), our model must also allow for a positive ATP yield when the physiological direction is methyl-THF oxidation. According to the model, H_2_ is converted by the bifurcating hydrogenase to additional 0.5 mol Fd_red_ and 0.5 mol NADH. The entire NADH is further used for Fd reduction by a reversal of the Rnf complex. Resulting Fd_red_ can subsequently be used for CO_2_ reduction in the carbonyl branch. During acetogenesis from methanol, 4 mol ATP are synthesized by substrate level phosphorylation, but for methyl group oxidation by the Rnf-MetVF complex, reverse electron flow is required, which is generated by hydrolysis of 0.56 ATP. Also, Fd reduction with NADH by the Rnf complex is driven by the H^+^ gradient; hence, 0.83 more ATP are hydrolyzed for generation of the gradient. In sum, 2.61 ATP are generated per 3 mol acetate (0.87 ATP/acetate) (Fig. 9c).

**Fig. 10:**
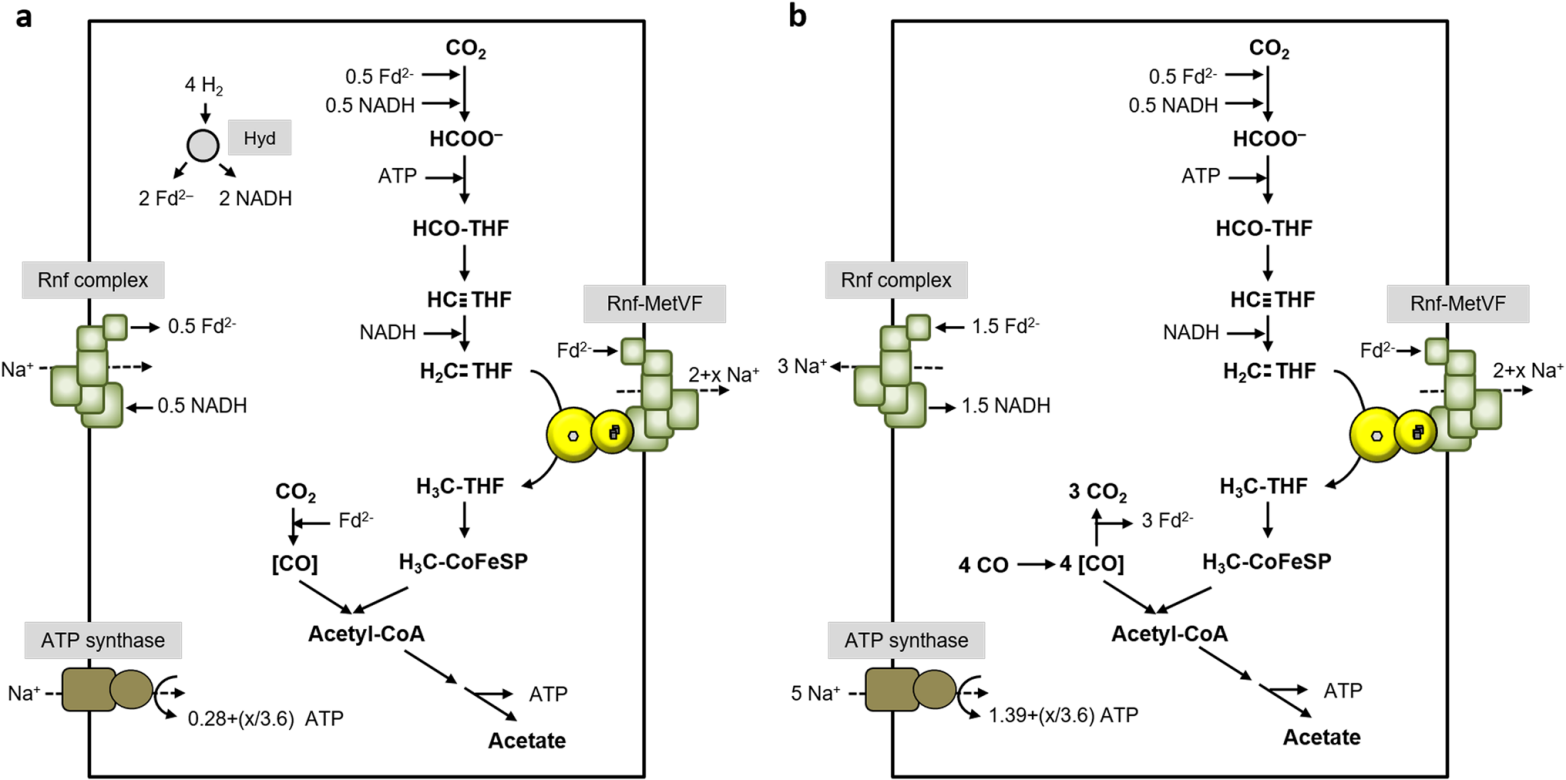
Model of Rnf-coupled methylene-THF reduction in acetogenesis of *C. aceticum.* Acetate is formed from H_2_ + CO_2_ (**a**) or CO (**b**). Acetogenesis from H_2_ + CO_2_ is feasible if we assume methylene-THF reduction by a membrane-coupled Rnf-MetVF complex. Assumed stoichiometries: H^+^/ATP = 3.6 (ATP synthase), 2 H^+^/2 e^−^ (Rnf) and 2 + x H^+^/2 e^−^ (Rnf-MetVF). ATP gain might be enhanced by x/3.6 ATP. For description, see text

In *C. aceticum* CO_2_ reduction is catalyzed by an electron-bifurcating formate dehydrogenase, which uses Fd_red_ and NADH as electron donors (Wiechmann and Müller 2021). The methylene-THF dehydrogenase is, like in *E. callanderi*, NADH dependent and the MTHFR is, as previously stated, of the MetVF-type. The Rnf complex as well as the ATP synthase were proven to be Na^+^ dependent as it is known for *A. woodii* (Wiechmann and Müller 2021). During autotrophic acetogenesis using H_2_ + CO_2_ as substrate, electrons are transferred from 4 mol H_2_ to Fd and NAD^+^ by an electron bifurcating hydrogenase. CO_2_ is reduced by 0.5 mol of Fd_red_ and NADH, respectively. Methylene-THF dehydrogenase uses 1 mol NADH for reduction of methenyl-THF. The remaining NADH is used for endergonic Fd reduction by the Rnf complex, driven by Na^+^ influx. Then, 1 mol ferredoxin is used for reduction of methylene-THF by the proposed Rnf-MetVF complex and CO_2_ in the carbonyl branch, respectively. In sum, 1 Na^+^ is left for the synthesis of 0.28 ATP by the ATP synthase (Fig. 10a). Acetogenesis from CO has a much higher ATP yield of 1.39 ATP (Fig. 10b).

## Conclusion

Although the type II-MTHFR uses Fd_red_ as reductant in vitro, this is to be excluded in vivo since the ATP yield of acetogenesis from H_2_ + CO_2_ becomes negative. The observed Fd_red_-dependent methylene-THF reduction in vitro may involve flavins, reduced by ferredoxin, as direct electron donors for the MTHFR. We present a hypothesis for a membrane-bound electron transport to methylene-THF by hooking up Rnf and Ech to the MTHFR. Such an electron transport chain would lead to chemiosmotic energy conservation and allows growth of both Rnf- and Ech acetogens on H_2_ + CO_2_ but also on other substrates. During growth on methanol, methyl-THF has to be oxidized to methylene-THF with concomitant reduction of ferredoxin. Our model also accounts for that by providing a solution to overcome the energetic barrier by reverse electron transport. This hypothesis can be tested to some extent in the future by biochemical analysis, but more important is the establishment of genetic techniques to prove/disprove our model by mutational studies.

## Supplementary Information

Below is the link to the electronic supplementary material.

Supplementary file1 (DOCX 161 KB)

## Data Availability

Not applicable.
