# Supplementary material for: Is reduced ferredoxin the physiological electron donor for MetVF-type methylenetetrahydrofolate reductases in acetogenesis? A hypothesis
Source: Int Microbiol. 2021 Jul 13;25(1):75–88. doi: 10.1007/s10123-021-00190-0 (PMC8760232; doi:10.1007/s10123-021-00190-0)
Supplement: Supplementary file 1 — Supplementary file1 (DOCX 161 KB) [file 10123_2021_190_MOESM1_ESM.docx]

**Supplementary information:**

Is reduced ferredoxin the physiological electron donor for MetVF-type methylenetetrahydrofolate reductases in acetogenesis?

A hypothesis

International Microbiology

Christian Öppinger^1*^, Florian Kremp^1*^ and Volker Müller ^1#^

*^1^Department of Molecular Microbiology & Bioenergetics, Institute of Molecular Biosciences, Johann Wolfgang Goethe University, Max-von-Laue Str. 9, D-60438 Frankfurt, Germany*

**These authors contributed equally to this study*

*#Corresponding author. Mailing address: Department of Molecular Microbiology & Bioenergetics, Institute of Molecular Biosciences, Johann Wolfgang Goethe University, Max-von-Laue-Str. 9, D-60438 Frankfurt, Germany. Phone: 49-69-79829507. Fax: 49-69-79829306. E-mail:* [*vmueller@bio.uni-frankfurt.de*](mailto:vmueller@bio.uni-frankfurt.de)*.*

Running title: The MetVF-type MTHFR


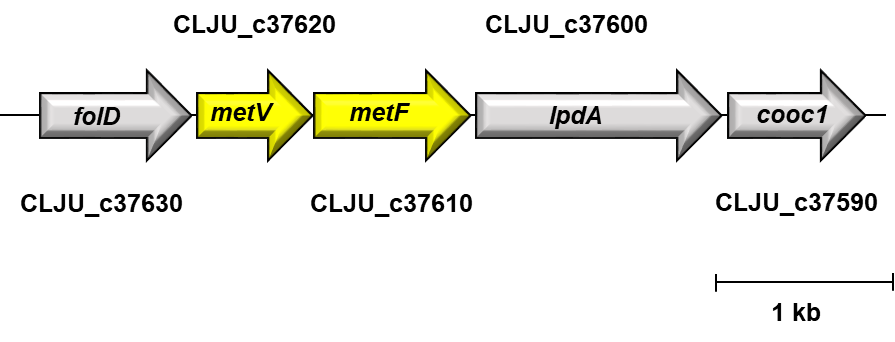


**Supplementary Figure 1 Genetic organization of the methylene-THF reductase in *C. ljungdahlii.*** The MTHFR encoding genes *metV* (CLJU_c37620) and *metF* (CLJU_c37610) are located downstream of *folD* (CLJU_c37630) that codes for a bifunctional methylene-THF dehydrogenase/methylene-THF cyclohydrolase. Downstream of *metF* a dihydrolipoamide dehydrogenase (*lpdA,* CLJU_c37600) and the nickel-insertion protein of the CODH/ACS (*cooC1,* CLJU_c37590) are encoded


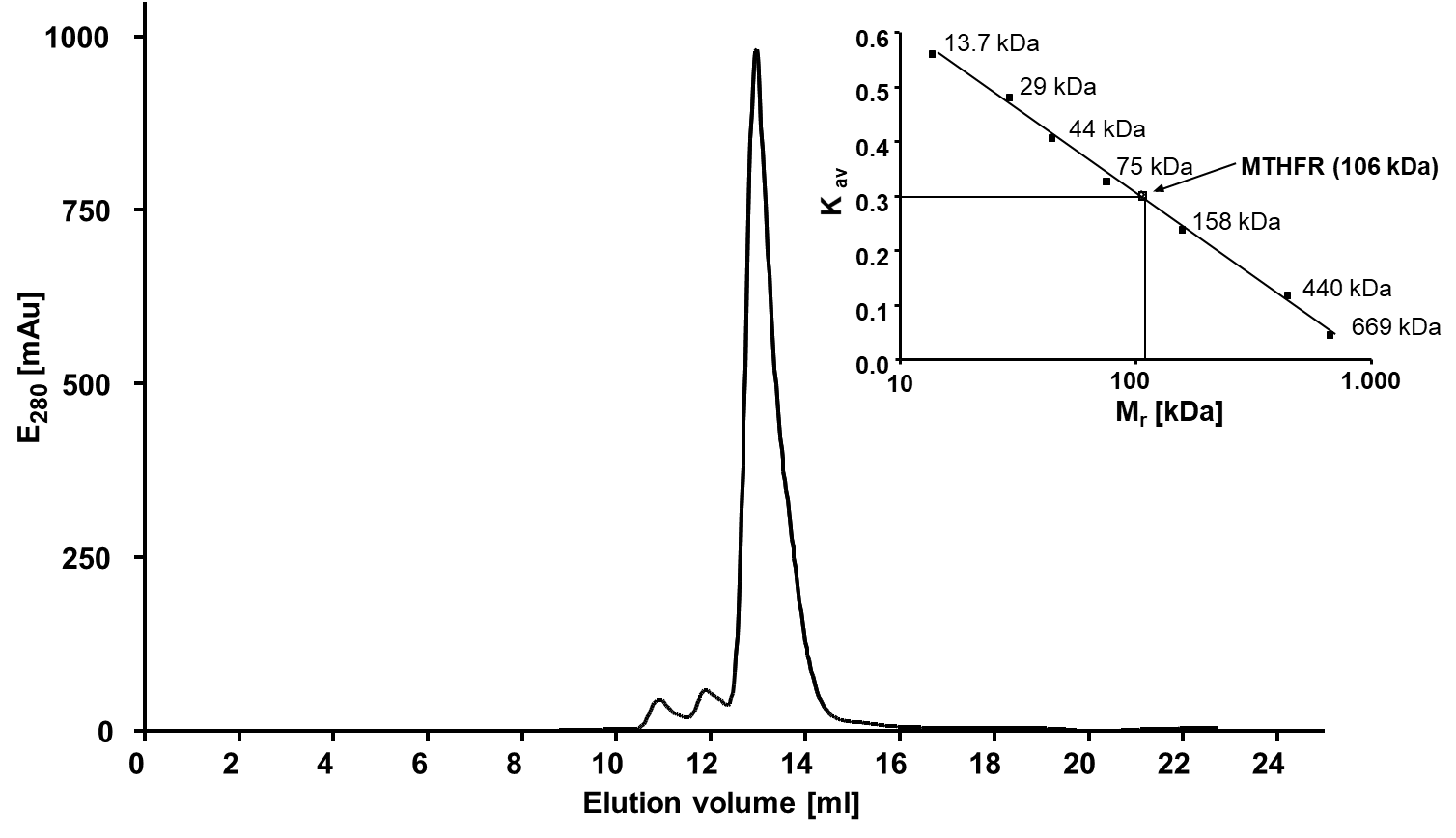


**Supplementary Figure 2 Molecular mass determination of the MTHFR.** The molecular mass of the MTHFR was determined by analytical gel filtration. The MTHFR was loaded onto a Superdex® 200 Increase column previously equilibrated with buffer D (50 mM Tris-HCl, 20 mM MgSO_4_, 20% glycerol, 250 mM NaCl 2 mM DTE, 4 µM resazurin, pH 7.6). Elution was performed with a flow of 0.2 ml/min. The molecular mass was calculated according to K_av_ = (V_e_ – V_0_)/(V_c_ - V_0)_ with V_0_ = 8.23 ml, V_c_ = 24 ml and V_e_ = 12.95 ml


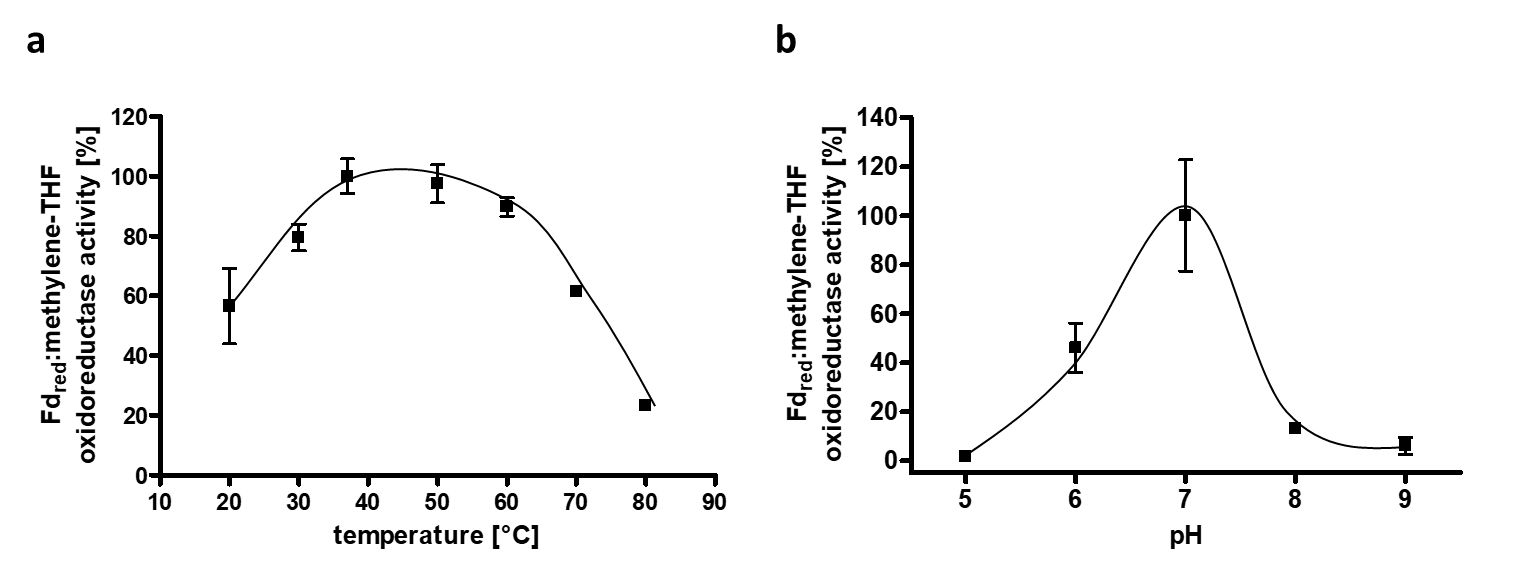


**Supplementary Figure 3 Influence of temperature and pH to the catalytic activity of the MTHFR.** To determine the influence of temperature, the MTHFR was measured in NaP_i_ buffer as described in materials and methods (a). To determine the influence of the pH a combined buffer was used and the measurements were performed at 24°C (b). 100 % activity correspond to 34.7 U/mg (a) and to 486 mU/mg (b). Each measurement was performed in duplicate


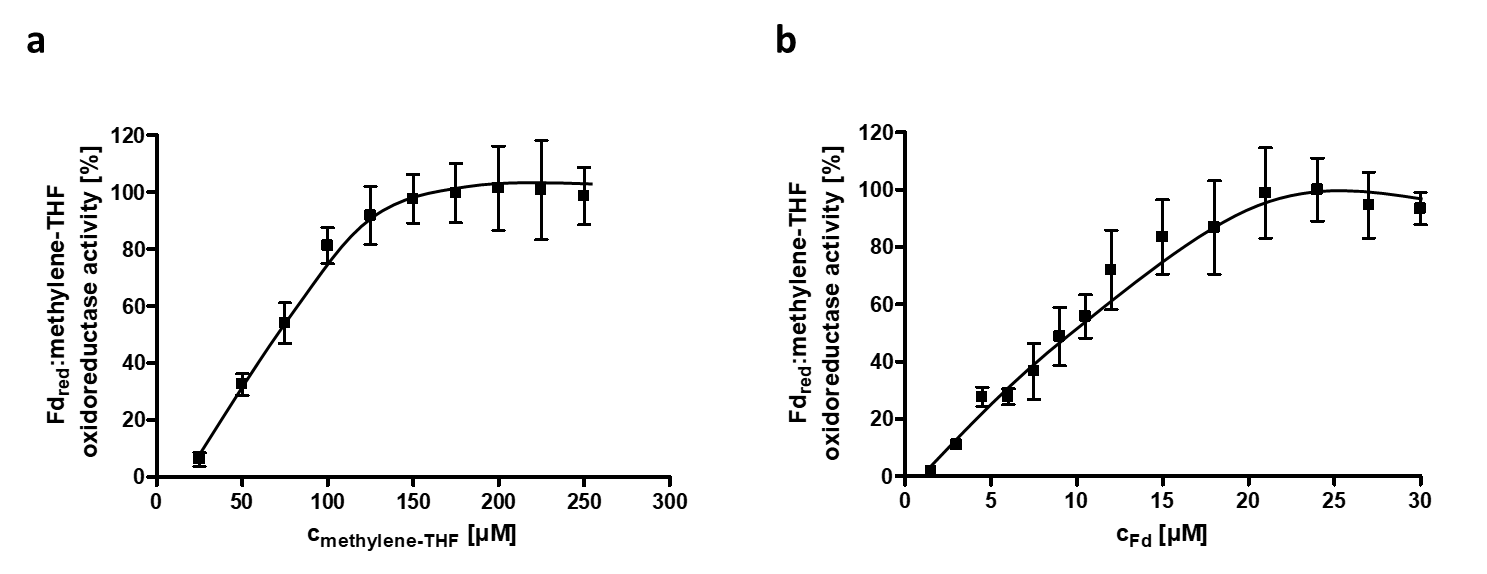


**Supplementary Figure 4 K_m_-value determination for methylene-THF and Fd_red_ in the Fd_red_:methylene-THF oxidoreductase activity of the MTHFR.** The measurements were performed in triplicates as described in materials and methods with different amounts of either methylene-THF (a) or Fd_red_ (b). 100 % activity correspond to 48.8 U/mg (a) and to 71.7 U/mg (b)
